# Characterization of Three New Insect-Specific Flaviviruses: Their Relationship to the Mosquito-Borne Flavivirus Pathogens

**DOI:** 10.4269/ajtmh.17-0350

**Published:** 2017-09-05

**Authors:** Hilda Guzman, Maria Angelica Contreras-Gutierrez, Amelia P. A. Travassos da Rosa, Marcio R. T. Nunes, Jedson F. Cardoso, Vsevolod L. Popov, Katherine I. Young, Chelsea Savit, Thomas G. Wood, Steven G. Widen, Douglas M. Watts, Kathryn A. Hanley, David Perera, Durland Fish, Nikos Vasilakis, Robert B. Tesh

**Affiliations:** 1 Department of Pathology and Institute for Human Infection and Immunity, University of Texas Medical Branch, Galveston, Texas;; 2 Programa de Estudio y Control de Enfermedades Tropicales – PECET – SIU – Sede de Investigacion Universitaria – Universidad de Antioquia, Medellin, Colombia;; 3 Center for Technological Innovation, Evandro Chagas Institute, Ministry of Health, Ananindeua, Para, Brazil;; 4 Postgraduate Program in Virology, Evandro Chagas Institute, Ministry of Health, Ananindeua, Para, Brazil;; 5 Department of Biology, New Mexico State University, Las Cruces, New Mexico;; 6 Department of Ecology and Evolutionary Biology, Yale University, New Haven, Connecticut;; 7 School of Public Health, University of Washington, Seattle, Washington;; 8 Department of Biochemistry and Molecular Biology, University of Texas Medical Branch, Galveston, Texas;; 9 U.S. Naval Medical Research Unit-6, Callao, Peru;; 10 Border Biomedical Research Center, University of Texas at El Paso, El Paso, Texas;; 11 Institute of Health and Community Medicine, Universiti Malaysia Sarawak, Kota Samarahan, Sarawak, Malaysia;; 12 Department of Epidemiology of Microbial Diseases, Yale School of Public Health, New Haven, Connecticut

## Abstract

Three novel insect-specific flaviviruses, isolated from mosquitoes collected in Peru, Malaysia (Sarawak), and the United States, are characterized. The new viruses, designated La Tina, Kampung Karu, and Long Pine Key, respectively, are antigenically and phylogenetically more similar to the mosquito-borne flavivirus pathogens, than to the classical insect-specific viruses like cell fusing agent and *Culex* flavivirus. The potential implications of this relationship and the possible uses of these and other arbovirus-related insect-specific flaviviruses are reviewed.

## INTRODUCTION

During the past two decades, there has been a dramatic increase in the discovery and characterization of novel insect-specific viruses (ISVs).^1–3^ This has coincided with advances in molecular tools for virus detection and the growing interest in insect microbiomes. Many of the new ISVs appear to be members of the family *Flaviviridae*, genus *Flavivirus,* and are common in insect populations in nature, with a worldwide geographic distribution.

The terms “insect-specific” or “insect-restricted” viruses in current usage generally refer to viruses that naturally infect hematophagous *Diptera* and that replicate in mosquito cells in vitro, but do not replicate in vertebrate cells or infect humans or other vertebrates.^1^ This is in contrast to the classical arthropod-borne viruses (arboviruses) that are maintained principally, or to an important extent, through biological transmission between susceptible vertebrate hosts by hematophagous arthropods.^4^ The arboviruses are dual host (vertebrate and arthropod) viruses, whereas the ISVs appear to involve only hematophagous insects.

To date (December 2016), approximately 35 insect-specific flaviviruses (ISFs) have been described.^1,3,5–12^ The ISFs can be separated into two distinct groups, based on their phylogenetic and antigenic relationships (Table 1). The first and largest group consists of the classical insect-specific flaviviruses (cISFs), such as cell fusing agent, *Culex* flavivirus (CxFV), and Kamiti River (KRV) viruses. The cISFs constitute a separate clade distinct from the vertebrate pathogenic flaviviruses. The second ISF group consists of the arbovirus-related or dual host affiliated insect-specific flaviviruses (dISFs).^3^ The dISFs are phylogenetically more similar to the flavivirus vertebrate pathogens than to the cISFs. Furthermore, as shown in this report, the dISFs are also closely related antigenically to some of the flavivirus pathogens, like West Nile (WNV), Zika, and dengue viruses. These similarities raise the possibility that some of the dISFs might modulate arbovirus infection and transmission in a dually infected mosquito host or that they could be useful in developing potential flavivirus vaccines or reagents.^1,3^

**Table 1:** Insect-specific flaviviruses isolated from or detected in hematophagous insects (*Culicidae* and *Psychodidae*)^1,5–12,45,46^

Classical insect-specific flaviviruses (cISFs)	Arbovirus-related insect-specific flaviviruses (dISFs)
*Aedes* cinereus flavivirus	Barkedji virus
*Aedes* flavivirus	Chaoyang virus
*Aedes* galloisi flavivirus	Donggang virus
Calbertado virus	Ilomantsi virus
Cell fusing agent virus	Lammi virus
*Culex* flavivirus	Marisma mosquito virus
*Culex* theileri flavivirus	Nanay virus
Czech *Aedes* vexans flavivirus	Nhumirim virus
Hanko virus	Nounane virus
Kamiti River virus	Aripo virus
Nakiwogo virus	La Tina virus
Nienokoue virus	Long Pine Key virus
Palm Creek virus	Kampung Karu virus
Quang Binh virus	Paraiso Escondido virus*
Mercadeo virus	
Culiseta flavivirus	
Yamadai flavivirus	
Parramatta River virus	
Mediterranean *Culex* flavivirus	
Assam virus	
Xishuangbanna flavivirus	
*Aedes* caspius flavivirus	
Anopheles flavivirus	
Phlebotomus-associated flavivirus*	

* Sandfly (*Psychodidae*) associated.

## MATERIALS AND METHODS

### Viruses (general).

A total of 31 ISFs were included in this study. Their names, strain designations, GenBank numbers, host source, and geographic origin are given in Table 2. More extensive information is given below on the three new ISFs. The viral sequences and supporting information on the vertebrate-pathogenic flaviviruses were obtained from GenBank and previous publications.

**Table 2:** GenBank accession numbers and information for 31 insect-specific flaviviruses used in phylogenetic analysis

Virus name	Strain designation	GenBank number	Host source	Geographic origin
Quang Binh (QBV)	VN180	NC_012671	*Culex tritaeniorhynchus*	Vietnam
Yunnan *Culex* flavivirus (YNCxFV)	LS FlaviV-A20-09	NC_021069	*Culex tritaeniorhynchus*	China
*Culex* theileri flavivirus (CTFV)	153	HE 574573	*Culex theileri*	Portugal
*Culex* flavivirus	Tokyo	NC_008604	*Culex pipiens*	Japan
Nakiwogo (NAKV)	Uganda 08	NC_030400	*Mansonia africana*	Uganda
Palm Creek (PCV)	56	KC 505248	*Coquillettidia xanthogastor*	Australia
Nienokoue (NIEV)	B51/C1/2004	NC_024299	*Culex* sp*.*	Ivory Coast
Mercadeo (MECDV)	ER-M10	NC_027819	*Culex* sp.	Panama
Kamiti River (KRV)	SR-82	NC_005064	*Aedes mcintoshi*	Kenya
*Aedes* flavivirus (Ae FV)	Narita-21	NC_012932	*Aedes albopictus*	Japan
Cell fusing agent (CFAV)	Original	NC_001564	*Aedes aegypti*	USA
Xishuangbanna (XFV)	XSBNAeFV	KU 201526	*Aedes albopictus*	China
Hanko (HANKV)	NA	NC-030401	Mosquito*	Finland
Nhumirim (NHUV)	BrMS-MQ10	NC-024017	*Culex chidesteri*	Brazil
Barkedji (BJV)	ArD86177	EU 078325	*Culex perexigus*	Senegal
Nounane (NOUV)	Nounane B3	EU 159426	*Uranotaenia mashonaensis*	Ivory Coast
Kampung Karu (KPKV)	SWK P44	KY320648	*Anopheles tessellatus*	Sarawak (Malaysia)
Long Pine Key (LPKV)	EVG 5-72	KY290256	*Anopheles crucians*	USA
Long Pine Key (LPKV)	EVG 1-33	KY290249	*Aedes atlanticus*	USA
Long Pine Key (LPKV)	EVG 1-42	KY290250	*Anopheles crucians*	USA
Long Pine Key (LPKV)	EVG 2-28	KY290251	*Aedes atlanticus*	USA
Long Pine Key (LPKV)	EVG 2-30	KY290252	*Aedes atlanticus*	USA
Long Pine Key (LPKV)	EVG 2-81	KY290253	*Aedes atlanticus*	USA
Long Pine Key (LPKV)	EVG 2-86	KY290254	*Aedes atlanticus*	USA
Long Pine Key (LPKV)	EVG 5-61	KY290255	*Culex nigripalpus*	USA
La Tina (LTNV)	49 LT 96	KY320649	*Aedes scapularis*	Peru
Marisma mosquito (MMV)	HU 4528/07	KY347801	*Aedes caspius*	Spain
Donggang (DGV)	DG 0909	JQ 086551	*Aedes* sp*.*	China
Ilomantsi (ILOV)	M0724	KC692067	Mosquito*	Finland
Lammi (LAMV)	M 0719	KC692068	*Aedes cinerus*	Finland
Chaoyang (CHAOV)	Deming	FJ 883471	*Aedes vexans*	China

* Genus and species not identified.

### New flaviviruses.

#### La Tina virus (LTNV).

strain 49 LT96, was isolated in C6/36 cells at the U.S. Naval Medical Research Unit Number 6 (NAMRU-6) in Lima, Peru, from a pool of female *Aedes scapularis* mosquitoes collected on August 22, 1996, in a horse-baited Shannon trap by M.R. Mendez. The collection site was surrounded by irrigated rice fields near the village of La Tina, Piura Province, Peru (latitude 04°24′, longitude 79°15′). After isolation at the NAMRU-6 facility, the virus was subsequently sent to the University of Texas Medical Branch (UTMB) for further study and characterization.

#### Long Pine Key virus (LPKV).

A total of eight isolates of LPKV were made in C6/36 cells at UTMB from mosquitoes collected in CDC-type light traps placed in various habitats at Long Pine Key (latitude 25°40′, longitude 80°66′) within Everglades National Park in southern Florida. Mosquito collections were made between June 13 and July 25, 2013, by a team from Yale University studying the distribution, abundance, and species composition of mosquitoes and mosquito-borne viruses occurring in the Florida Everglades. Mosquito collections were approved by the U.S. National Park Service under Collecting Permit EVER-2013-SCI-0032. Table 2 lists the strain designations and mosquito host species of the eight LPKV isolates. LPKV strain 5-72, obtained from a pool of 50 *Anopheles crucians* collected on July 24, 2013, was designated as the prototype.

#### Kampung Karu virus (KPKV).

strain SWK P44, was isolated from a single female *Anopheles tesselatus* mosquito collected in a gravid mosquito trap (Bioquip 2800) on October 16, 2013, in the village of Kampang Karu, Kuching District, Sarawak, Malaysia (latitude 1°17′, longitude 110°16′). KPKV was originally isolated in C6/36 cell cultures at the University Malaysia Sarawak and was sent to UTMB for further study and characterization.

### Transmission electron microscopy.

For ultrastructural analysis in ultrathin sections infected cells were fixed for at least 1 hour in a mixture of 2.5% formaldehyde prepared from paraformaldehyde powder, and 0.1% glutaraldehyde in 0.5 M cacodylate buffer pH 7.3 to which 0.01% picric acid and 0.03% CaCl_2_ were added. The C6/36 monolayers were washed in 0.1 M cacodylate buffer, cells were scraped off and processed further as a pellet. The pellets were postfixed in 1% OsO_4_ in 0.1 M cacodylate buffer pH 7.3 for 1 hour, washed with distilled water and *en bloc* stained with 2% aqueous uranyl acetate for 20 minutes at 60°C. The pellets were dehydrated in ethanol, processed through propylene oxide, and embedded in Poly/Bed 812 (Polysciences, Warrington, PA). Ultrathin sections were cut on Leica EM UC7 μLtramicrotome (Leica Microsystems, Buffalo Grove, IL), stained with lead citrate and examined in Philips 201 transmission electron microscope (Philips Electron Optic, Eindhoven, Netherlands) at 60 kV.

### Preparation and use of immune sera.

Since the ISFs by definition do not infect vertebrates or vertebrate cells, we were unable to produce ISF-specific antibodies. Attempts to immunize mice with ISF antigens produced from infected C6/36 cells inevitably resulted in antibodies that reacted with uninfected C6/36 cell controls. Attempts to absorb out the mosquito cell antibodies were unsuccessful; consequently, we used heterologous mouse hyperimmune ascitic fluids (MIAFs), prepared with infected mouse brain antigens of selected flavivirus pathogens, such as WNV, dengue type-2, Zika, yellow fever, and Japanese encephalitis viruses, in serologic tests. These antibodies were obtained from the World Reference Center for Emerging Viruses and Arboviruses; their homologous titers, as determined by hemagglutination-inhibition (HI) tests, are given in Table 3. Methods used to prepare these MIAFs, using mouse brain antigens, have been described before.^13^ All animal work and preparation of murine antibodies was covered by an approved UTMB Institutional Animal Care and Use Committee protocol (number 9505045).

**Table 3:** Results of hemagglutination-inhibition tests with hyperimmune flavivirus antibodies and C6/36 antigens of selected mosquito-specific flaviviruses

Antibody	Antigen				
	La Tina	Marisma	Nanay [45]	Aripo [46]	Homologous
Dengue 1	–	80	40	20	320
Dengue 2	–	160	80	80	640
Dengue 3	160*	80	80	80	320
Dengue 4	0	80	160	20	1,280
Japanese encephal.	80	–	160	320	5,120
St. Louis encephal.	320	–	–	320	5,120
West Nile	320	2,560	640	2,560	5,120
Usutu	0	640	–	–	640
Rocio	80	–	–	160	5,120
Ilheus	320	–	640	2,560	2,560
Yellow fever	–	80	20	–	640
Zika	0	40	–	–	640

* Reciprocal of highest antibody titer giving positive result.

0 = < 20—–indicates not tested.

### Immunofluorescent studies.

The antigens used in immunofluorescent studies were ISF-infected C6/36 cells. Six or 7 days after virus inoculation, the infected cells were scraped from the surface of the culture flask and spotted onto Cel-Line 12-well glass slides (Thermo Fisher Scientific, Waltham, MA) for examination by indirect fluorescent antibody test (IFAT),^14^ using the heterologous mouse hyperimmune polyclonal antibodies described above at a 1:20 dilution.

### Preparation of ISF antigens for HI tests.

In preliminary studies,^15^ we found that it was possible to prepare serologic antigens for some ISFs, using infected C6/36 cells. Selected ISFs were inoculated into flask cultures of C6/36 cells maintained at 28°C, as noted above. When most of the cells showed viral cytopathic effect (CPE), the entire flask, containing medium and cells, was frozen at −80°C. For preparation of an acetone-extracted antigen, the flask contents were thawed and dropped through a 26 gauge needle into 20 volumes of chilled acetone. Within 5 minutes, this mixture was centrifuged at 1,000 rpm for 2 minutes and the supernatant fluid discarded. The sediment was resuspended in another 20 volumes of chilled acetone, shaken, and held for 1 hour at 4°C. The mixture was then centrifuged at 1,600 rpm for 5 minutes, the acetone decanted and the sediment dried for 1 hour by vacuum. The dried sediment was rehydrated in a volume of borate saline solution pH 9.0 equal to the original fluid used for extraction and was stored frozen at −80°C until used.

### HI tests.

A standard HI test was done in microtiter plates, according to methods described previously.^16^ Nonspecific inhibitors in the antisera were acetone extracted by the method of Clarke and Casals.^17^ The ISF antigens (infected C6/36 cells) were described above. Antibodies (MIAFs prepared to various flavivirus pathogens) were tested at serial 2-fold dilutions from 1:20 to 1:5120 at pH 6.0–6.2 with 4 units of antigen and a 1:200 dilution of goose erythrocytes, following established protocols.^16^

### Evaluation of growth of LTNV, LPKV, and KPKV in mosquito and mammalian cells.

To determine the host range of the three new ISFs, their replication was studied in the following cell lines: *Aedes albopictus*, clone C6/36 (CRL-1660); baby hamster kidney, BHK-21 (CCL-10); and African green monkey kidney, Vero E6 (CRL-1586). The three cell lines were originally obtained from the American Type Culture Collection (ATCC), Manassas, VA. The mosquito cells were maintained at 28°C and the vertebrate cells at 37°C in 12.5 cm^2^ tissue culture flasks with 5 mL of medium recommended in the ATCC specification sheets. When a confluent monolayer of cells was present, 200 uL of a stock of each virus (LTNV, LPKV, and KPKV) were inoculated into flasks of C6/36, BHK-21, and Vero E6 cells. After incubation for 2 hours, each flask was washed three times with 5 mL of maintenance medium, with aspiration to remove all remaining medium between washes. The medium was replaced and a sample (500 uL) taken as a day 0 control. Each day thereafter, for 7 consecutive days, the medium was completely removed, sampled, and fresh medium added to each flask, as described above. The daily samples (day 0–7) were subsequently assayed by reverse transcription-polymerase chain reaction (RT-PCR). Primer sequences available upon request.

### Evaluation of growth in suckling mice.

One litter of 1- to 2-day-old Institute for Cancer Research (ICR) mouse pups (*N* = 10) were inoculated intracranially with approximately 15 uL of C6/36 culture fluid from stocks of each of the three ISVs (LTNV, LPKV, and KPKV). After inoculation, the pups were returned to their dams and were examined daily for 14 days for signs of illness or death. Mice were purchased from Harlan Sprague–Dawley (Indianapolis, IN); this animal work at UTMB was carried out under IACUC-approval protocol number 9505045.

### Extraction of viral RNA.

For all ISVs, fluid supernatant from cultures of infected C6/36 cells were used for RNA extraction and sequencing. Supernatants were harvested and clarified by low speed centrifugation (2,000 × *g*, 10 minutes at 4°C) once CPE was advanced. One milliliter of clarified supernatant from each virus was treated with a cocktail of DNases (14 U Turbo DNase [Ambion, Austin, TX], 20 U Benzonase [EMD Millipore, Billerica, MA], and 20 U RNase One [Promega, Madison, WI]) for 1 hour at 37°C. Viral RNA was then extracted using Trizol and resuspended in 50 μL RNase/DNase and protease-free water (Ambion, Austin, TX).

### Next generation sequencing.

Viral RNA (∼0.9 µg) was fragmented by incubation at 94°C for 8 minutes in 19.5 μL of fragmentation buffer (Illumina 15016648). A sequencing library was prepared from the sample RNA using an Illumina TruSeq RNA v2 kit following the manufacturer’s protocol. The sample was sequenced on a HiSeq 1500 using the 2 × 50 paired-end protocol. Reads in fastq format were quality-filtered, and any adapter sequences were removed, using Trimmomatic software.^18^ The *de novo* assembly program ABySS^19^ was used to assemble the reads into contigs, using several different sets of reads, and k values from 20 to 40. In all samples, host reads were filtered out before *de novo* assembly. The longest contigs were selected and reads were mapped back to the contigs using bowtie2^20^ and visualized with the Integrated Genomics Viewer^21^ to verify that the assembled contigs were correct. A total of 28.8, 10.0, 5.8, 8.5, 11.4, 9.5, 11.1, 16.5, 11.0, 19.9, and 21.0 million reads were generated for the samples containing MMV, KKV, LTNV, EVG 1_33, EVG 1_42, EVG 2_28, EVG 2_30, EVG 2_81, EVG 2_86, EVG 5_61, and EVG 5_72, respectively. Reads mapping to the virus in each sample comprised ∼1,960,000 (6.83%), ∼340,000 (3.37%), ∼350,000 (6.0%), ∼3,100,000 (36.6%), ∼3,300,000 (29.0%), ∼7,870,000 (82.6%), ∼460,000 (4.1%), ∼3,770,000 (22.8%), ∼2,780,000 (25.3%), ∼2,150,800 (10.8%), and ∼6,400,000 (30.5%), respectively.

### Molecular analyses and phylogenetics studies.

The evolutionary history was inferred by using the maximum likelihood method based on the General Time Reversible model. The tree with the highest log likelihood (−366425.2857) is shown. The percentage of trees in which the associated taxa clustered together is shown next to the branches. Initial tree(s) for the heuristic search were obtained automatically by applying Neighbor-Join and BioNJ algorithms to a matrix of pairwise distances estimated using the maximum composite likelihood approach, and then selecting the topology with superior log likelihood value. A discrete gamma distribution was used to model evolutionary rate differences among sites (5 categories [+G, parameter = 0.8309]). The rate variation model allowed for some sites to be evolutionarily invariable ([+I], 10.5704% sites). The tree is drawn to scale, with branch lengths measured in the number of substitutions per site. The analyses involved 93 nucleotide sequences. Codon positions included were 1st + 2nd + 3rd + Noncoding. All positions containing gaps and missing data were eliminated. There were a total of 5,981 positions in the final dataset. Evolutionary analyses were conducted in MEGA7.^22^

## RESULTS

### Isolation and culture results.

LTNV, LPKV, and KPKV were each initially isolated in cultures of C6/36 cells. In this cell line, the three viruses produced a similar CPE (rounding and detaching of cells from flask surface) 6 or 7 days after inoculation. Subsequent inoculation of the C6/36 culture fluid into BHK-21 or Vero E6 cells failed to produce detectable CPE. Likewise, intracranial inoculation of the infected C6/36 culture fluid into newborn ICR mice failed to produce illness or death in the animals.

To determine if LTNV, LPKV, and KPKV replication occurred in vertebrate cells without producing CPE, additional experiments were carried out in C6/36, Vero, and BHK cell cultures to assay for virus replication by RT-PCR. Samples of medium from the three ISV-inoculated cell lines were collected from day 0 to day 7, as described in the Methods section. After RNA extraction, a partial region of the following genes of each virus was amplified and run on gels: LTNV, a partial region covering part of the NS1 and NS2B genes with expected band size between 350 and 400 nucleotides (nt) long; KPKV, a partial region between the NS1 and NS2A genes with expected band size 500–600 nt long; and LPKV, a partial region of the NS3 gene with expected band size between 450 and 500 nt long (primer sequences available upon request). RNA extracted from culture fluid from the C6/36 cells infected with LTNV, LPKV, and KPKV from day 0 to day 7 postinoculation (dpi) displayed strong bands on all days (data not shown). In contrast, extracted and amplified viral RNA from the Vero and BHK cells inoculated with the three viruses showed decreasing intensity of the RNA bands from day 0 to day 7, indicating that the three new flaviviruses did not replicate in the two vertebrate cell lines (data not shown).

### Ultrastructure LTNV, LPKV, and KPKV.

**Figure 1.**
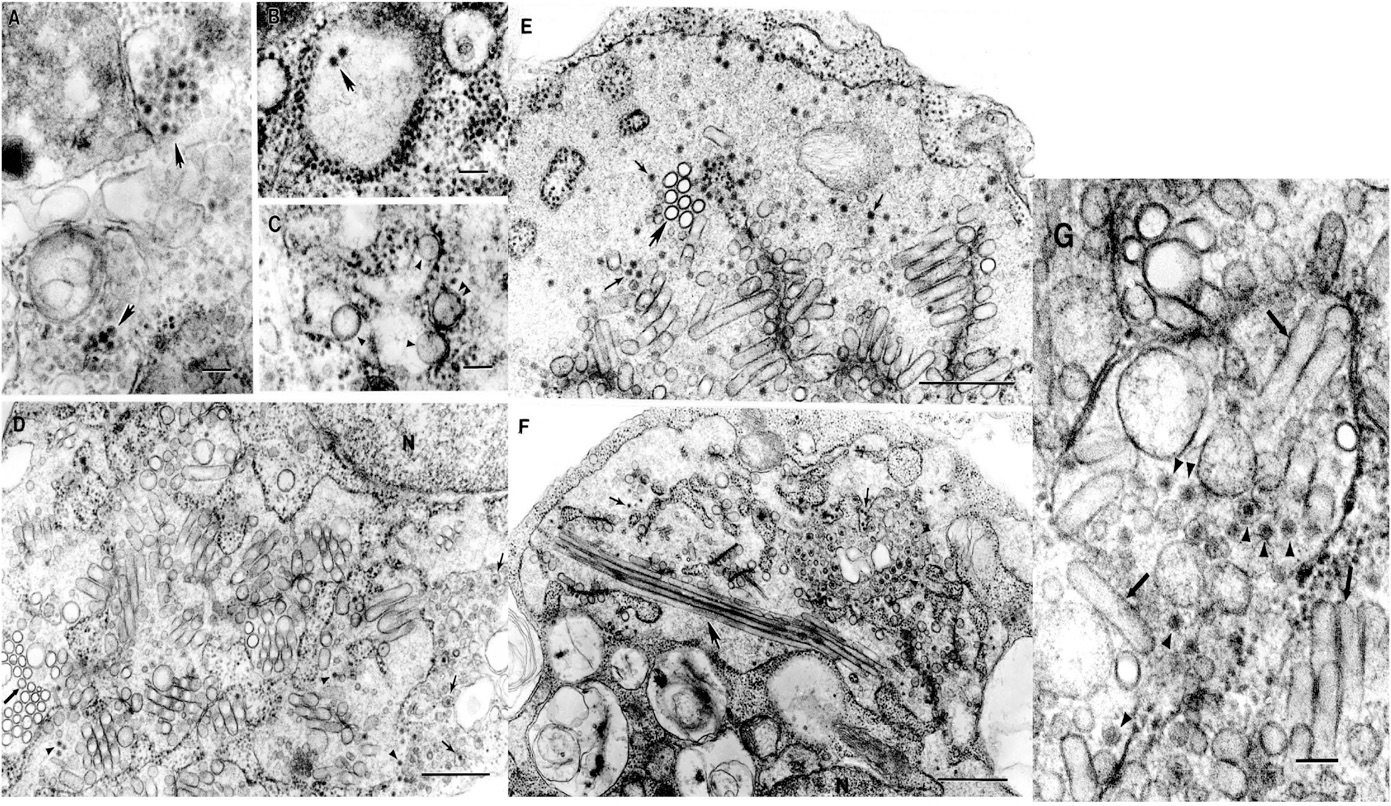
Ultrastructure of Kampung Karu (KPKV), Long Pine Key (LPKV) and La Tina (LTNV) viruses in C6/36 cells. (**A**) KPKV. Accumulations of virions ∼40 nm in diameter inside cytoplasmic vacuoles (arrows). Bar = 100 nm. (**B**) KPKV. Two virions inside a cistern of granular endoplasmic reticulum (arrow). Bar = 100 nm. (**C**) KPKV. Smooth membrane structures (SMS) inside an expanded cistern of granular endoplasmic reticulum (arrowheads) or in a tightly apposed cistern (double arrowhead). Bar = 100 nm. (**D**) LPKV. Virions (arrowheads) and SMS inside an enormous expansion of a cistern of granular endoplasmic reticulum. Thick arrow indicates cross sections of the SMSs. Virions can also be found inside individual small vacuoles (thin arrows). N-fragment of host cell nucleus. Bar = 0.5 µm. (**E**) LPKV. Virions (thin arrows) and SMS inside an enormous expansion of granular endoplasmic reticulum. Thick arrow indicates cross sections of SMSs. Bar = 0.5 µm. (**F**) LPKV. Virions (thin arrows) and SMS inside an enormous expansion of granular endoplasmic reticulum. Virions can be also observed inside individual vacuoles (arrowheads). Some SMS can be very long, up to 2.2 µm (thick arrow). N-fragment of the host cell nucleus. Bar = 0.5 µm. (**G**) LTNV. Virions (arrowheads) and SMS (arrows) inside an expanded cistern of granular endoplasmic reticulum. Bar = 100 nm.

In ultrathin sections of infected C6/36 cells, the three viruses had typical flavivirus morphology with intracellular localization and formation of smooth membrane structures (SMS) (Figure 1A–G). Virions were ∼40 nm in diameter and were localized mostly in cisternae of granular endoplasmic reticulum or inside vacuoles, in clusters (Figure 1A) or individually (Figure 1D, F, and G). In the cells infected with LPKV, granular endoplasmic reticulum could be enormously expanded and contained multiple SMS and virions (Figure 1D, E, and F). In some sections, these SMS could be unusually long, up to 2.2 µm (Figure 1F).

### HI tests.

In preliminary studies, we observed that polyclonal MIAFs prepared against selected flavivirus pathogens reacted in HI tests with some of the dISFs, using antigens prepared from infected C6/36 cell cultures. However, for most of the dISFs, we were unable to prepare reactive hemagglutinins. In the case of the three new ISFs (LTNV, LPKV, and KPKV), LTNV was the only virus that produced a reactive hemagglutinin.

Table 3 shows results of HI tests with MIAFs prepared against 12 mosquito-borne flavivirus pathogens and four antigens prepared from C6/36 cells infected with selected dISFs, including LTNV. Most of the MIAFs reacted with the four dISFs antigens tested, although the titers were lower than with the homologous antigens. The WNV antibody was the most reactive MIAF, with titers to Marisma and Aripo virus antigens just 2-fold lower (1:2560) than with the homogous (WNV) antigen (1:5120). The HI titer of Ilheus virus MIAF with Aripo virus antigen was equal (1:2560) to that with the homologous antigen. No reactions were observed in HI tests with the same MIAFs, using C6/36 antigens prepared against LPKV, KPKV, Mercadeo (MECDV), KRV, CxFV, Quang Binh virus, or uninfected (control) C6/36 cell antigens (data not shown).

### Indirect immunofluorescent tests.

**Figure 2.**
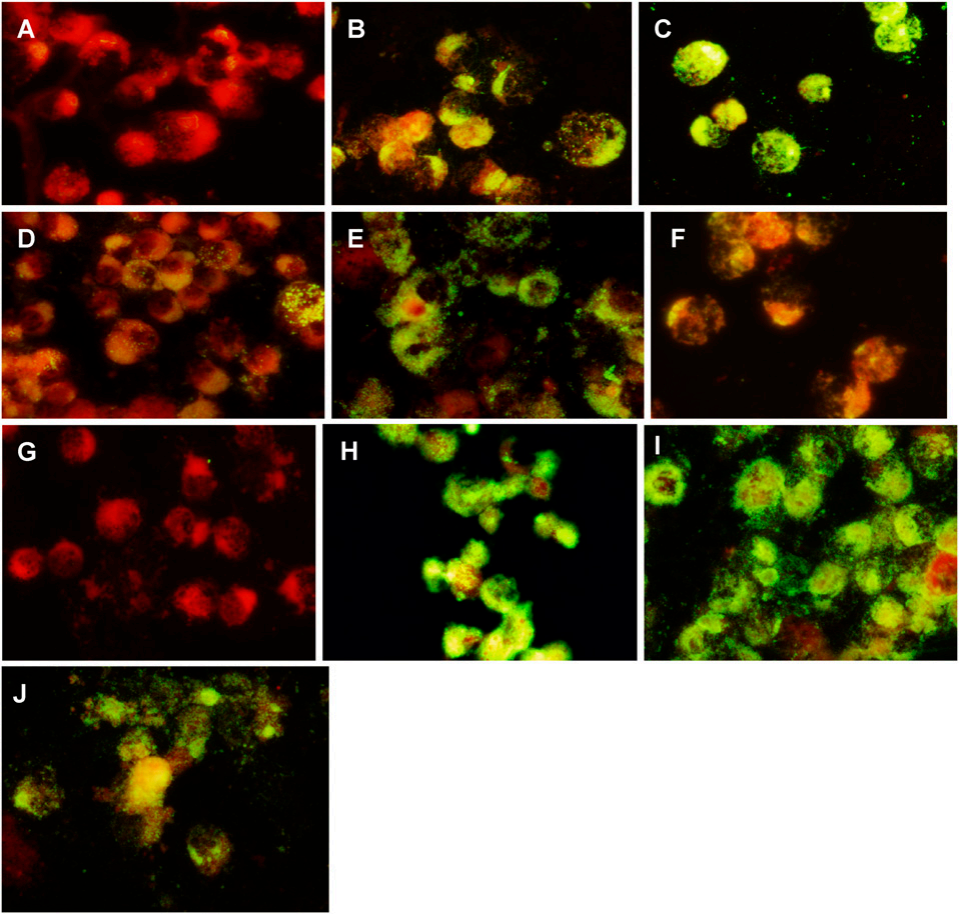
Results of indirect fluorescent antibody tests with C6/36 cells infected with various insect-specific viruses (antigen), using heterologous hyperimmune polyclonal antibodies prepared against West Nile (WNV), Dengue-2 (DENV-2) and Zika (ZIKV) viruses. Antibody dilutions 1:20. (**A**) Mercadeo virus X WNV antibody. (**B**) Long Pine Key virus X WNV antibody, (**C**) Marisma mosquito virus X WNV antibody. (**D**) La Tina virus X WNV antibody. (**E**) Nanay virus X ZIKV antibody. (**F**) Nhumirim virus X DENV-2 antibody. (**G**) Uninfected (control) C6/36 cells X WNV antibody. (**H**) WNV X WNV antibody. (**I**) DENV-2 X DENV-2 antibody. (**J**) ZIKV X ZIKV antibody.

Figure 2 illustrates the degree of immunofluorescence observed in IFATs done on C6/36 cells infected with six different ISFs; using WNV, dengue type 2, or Zika virus MIAFs diluted 1:20. Figure 2A shows MECDV-infected mosquito cells tested with WNV antibody. There is only a trace of fluorescence in this antigen–antibody reaction. Similar results were obtained in IFATs using two other cISFs (CxFV and KRV) (not shown). In contrast, Figure 2B–D shows results obtained with C6/36 cells infected with LPKV, Marisma mosquito, and LTNV, respectively, when tested with WNV antibody at the same dilution. The degree of fluorescence varied with each antigen; but as observed in HI tests (Table 2), Marisma mosquito virus antigen reacted intensely with WNV antibody. Figure 2E shows C6/36 cells infected with Nanay virus and tested with Zika virus MIAF at 1:20 dilution. Figure 2F shows C6/36 cells infected with Nhumirim virus and tested with dengue type 2 virus MIAF at 1:20 dilution. In each case, the five dISFs gave clear positive reactions, whereas the cISFs did not. The dISFs also reacted in IFATs with MIAFs prepared to some other flavivirus pathogens (i.e., dengue type 1, Ilheus, St. Louis encephalitis, and Japanese encephalitis), but the degree of fluorescence was less intense and is not shown. Figure 2G, shows uninfected C6/36 cells with DENV-2 MIAF at 1:20 dilution. Figure 2H–J shows WNV, DENV-2, and ZIKV infected C6/36 cells with their corresponding MIAFs. No fluorescence was observed in uninfected (control) C6/36 cells with any of the other flavivirus MIAFs used in this study (data not shown). The cytoplasm and nucleus of the mosquito cells both stain red with the Evan’s Blue counterstain that was used in our IFATs.

### Molecular analyses and phylogenetic studies.

**Figure 3.**
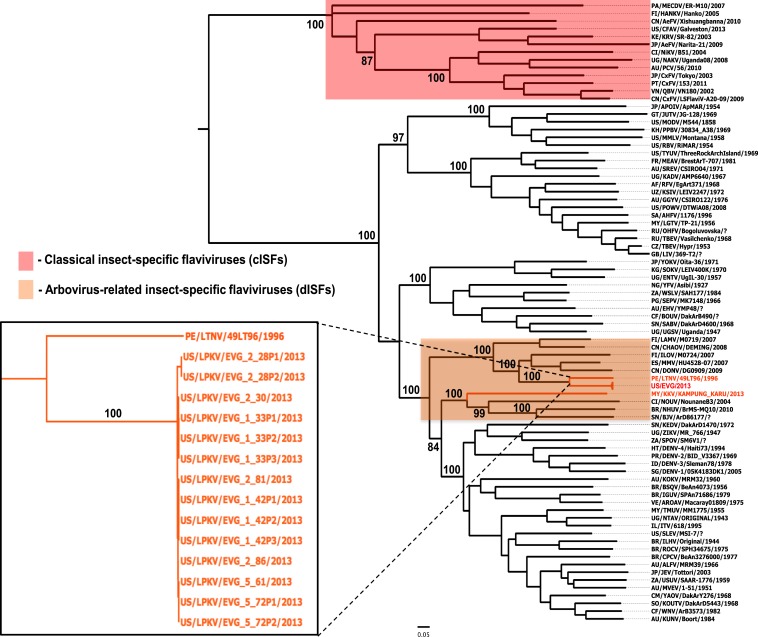
Maximum-likelihood (ML) phylogenetic tree of representative members of the genus *Flavivirus*. Highlighted in red are classical insect-specific flaviviruses (cISFs) and in orange are the arbovirus-related insect-specific flaviviruses (dISFs). Insert: Close-up of the dISFs described in this study. Bootstrap values are shown for most clades. All horizontal branch lengths are drawn to scale bar 0.05 nucleotide substitutions per site. The tree is midpoint-rooted for purposes of clarity only.

The evolutionary history of LKPV, KPKV, and LTNV viruses was reconstructed where their complete open reading frame (ORF) sequences were aligned to a dataset of sequences representing 93 flavivirus species. A consensus tree was obtained based on maximum-likelihood with bootstrap resampling of 1,000 replicates used to obtain confidence limits on individual branches. As expected the cISFs, represented by cell fusing virus (CFAV), KRV, CxFV, and others, clustered in a clade basal to all other member species of the *Flavivirus* genus (Figure 3). On the other hand, LKPV, KPKV, and LTNV group within the arbovirus-related insect-specific flavivirus (dISF) clade. Specifically, the eight LPKV isolates and LTNV virus are closely related to each other. On the other hand, KPKV has a close relationship with Nounane (NOUV), Barkedji (BJV), and Nhumirim (NHUV) viruses (100% bootstrap support).

### Genome organization of LTNV, LPKV, and KPKV.

**Figure 4.**
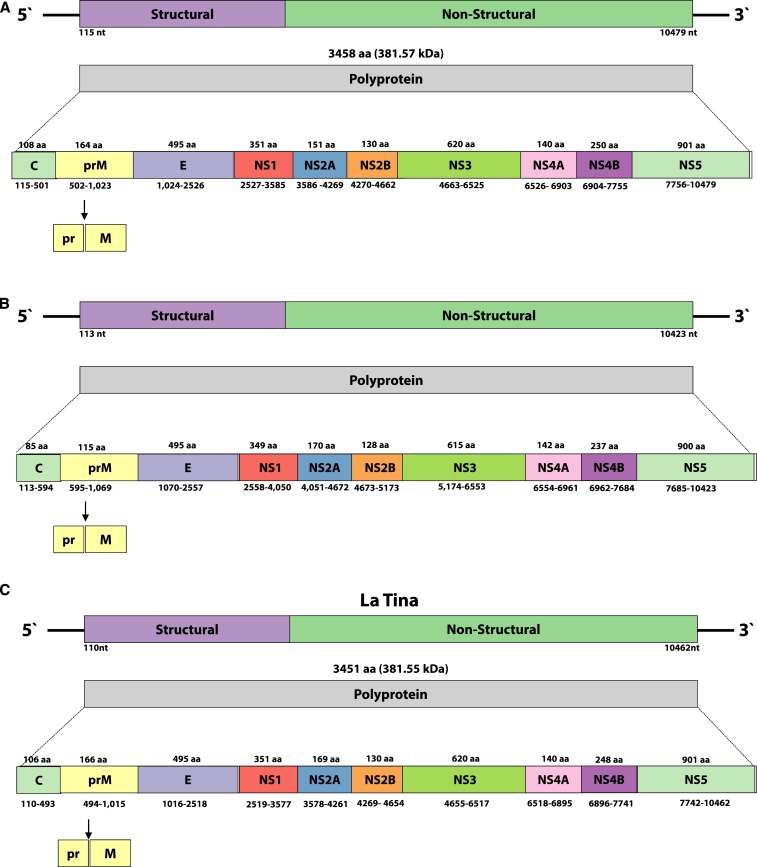
Genomic organization of Long Pine Key (**A**), Kampung Karu (**B**) and LaTina (**C**) viruses. Long grey boxes represent the polyproteins that after the cotransduction cleavage process form the three structural (C = capsid; prM = pre membrane; E = envelope) and seven non-structural (NS) proteins (NS1 to NS5) (represented by colored boxes). Numbers under the boxes correspond to the polyprotein genes. Start and stop codons expressed in nucleotides (nt) over the boxes represent the functional proteins, excluding the cleavage sites, and are expressed in aminoacids (aa). Arrow indicates the cleavage junction for pr and M proteins.

The size of the positive sense, near complete single-strand genomes of the three identified viruses are 10,859, 10,968, and 10,882 nucleotides (nt) long, for LPKV (KY290256), KPKV (KY320648), and LTNV (KY320649), respectively. A single ORF of 10,365 (3,454 aa), 10,311 (3,437 aa), and 10,356 (3,452 aa) nt for LPKV, KPKV, and LTNV, respectively, are flanked by untranslated regions at the 5′ and 3′ ends (Figure 4).

## DISCUSSION

The results of this study confirm that the ISFs can be divided into two distinct groups.^3^ The first and most numerous group is the cISVs (this group includes viruses such as CFAV, KRV, Calbertado, CxFV, and Palm Creek (Table 1). In the flavivirus phylogenetic tree shown in Figure 3, the cISVs form a divergent branch from the main tree. The cISVs also show little or no antigenic relationship with the flavivirus vertebrate pathogens in the main tree.

The second ISF group consists of the arbovirus-related or dISFs (it includes the three new viruses described in this report LPKV, LTNV and KPKV) as well as other agents such as NHUV, Marisma mosquito, and Donggang viruses (Table 1). The dISFs are genetically more similar to the mosquito-borne flavivirus pathogens, such as WNV, Ilheus, dengue, Zika, and Japanese encephalitis viruses, than they are to the cISFs (Figure 3). Some of the dISFs are also antigenically related to these same mosquito-borne flavivirus pathogens (Table 3 and Figure 2). Unfortunately, we were unable to test the antigenic relatedness of all the named dISFs, because some were not available to us and others have never been isolated. A number of the ISFs are known only from sequences obtained by RT-PCRs or from metagenomics studies. In addition, we were unable to produce reactive hemagglutinins for all of the available dISFs. Despite these limitations, the results of our limited serologic studies indicate that the dISFs are both genetically and antigenically closely related to important mosquito-borne flavivirus pathogens.

What are the possible implications of this relationship between the ISFs and important mosquito-borne flavivirus pathogens? One possible implication is that the ISFs, and the dISFs in particular, may alter the vector competence of their dipteran hosts. Since most of the ISFs described to date have been associated with mosquitoes, the following discussion will focus on the mosquito-specific flaviviruses.

The available data on the ISFs indicate that they are relatively common in mosquito populations in nature and that some, like CFAV and CxFV, have a wide geographic distribution.^1–13,23,24^ In addition, flavivirus-derived endogenous elements (EVEs) or nonretroviral integrated RNA viruses have been reported in the genomes of several mosquito species.^25–28^ ISFs and EVEs are part of the microbiome of many species and genera of mosquitoes, but they appear to have no obvious deleterious effect on their natural insect hosts.^1–3^ The available evidence suggests that the ISFs are maintained in mosquito populations by vertical transmission, and that they do not use vertebrate hosts as part of their life cycle, like most of the classical arthropod-borne viruses of vertebrates (arboviruses).^24,29^

There has been considerable speculation, but conflicting experimental evidence, that infection of a mosquito with an ISF can alter the insect’s vector competence for certain mosquito-borne flavivirus pathogens, due to heterologous interference. But most of the experimental studies of this phenomenon to date have used cISFs, such as CxFV or Palm Creek viruses; and the results have been mixed. ^1,30–35^ Some studies have suggested that coinfection reduces vector competence^30,32–35^ whereas others have found that it had no effect.^1,31^

A second problem is that most of the experimental studies have been done in vitro, using the C6/36 mosquito cell line. However, the C6/36 cell line has a dysfunctional antiviral RNA interference (RNAi) response,^31,36^ so in vitro results of dual infection may not be indicative of what actually occurs in a live mosquito with a functional RNAi response (in vivo). One study^34^ with *Culex* quinquefasciatus infected with Nhumirim virus and then challenged with WNV indicated that the dually infected mosquitoes were less competent vectors of WNV than control mosquitoes infected with WNV alone, but additional studies of dual infection with dISF and related flavivirus pathogens are needed in *live* mosquitoes to clarify this possibility. If a dISF reduced the vector competence of *Aedes aegypti* for Zika or dengue viruses, for example, potentially it might be used as a disease control agent, as some strains of *Wolbachia* have been used.^37^ If the candidate dISF was also vertically transmitted in mosquitoes, then theoretically it would be maintained in the vector population.

Another potential application for the dISVs could be to use them as platforms for development of vaccines or diagnostics.^1^ The insect-specific alphavirus, Eilat,^38^ is defective for vertebrate cell infection^39^ and has been exploited by using recombinant DNA technology to generate Eilat chimeras, where the structural polyprotein ORF was swapped with that of a vertebrate-pathogenic alphavirus to generate a chimera that is structurally indistinguishable from the pathogenic virus.^1^ Eilat-based alphavirus chimeras have been developed as vaccines for chikungunya and Venezuelan equine encephalitis viruses.^1^ Chimeras between Eliat and chikungunya viruses have also been shown to serve as high-quality antigens for enzyme-linked immunosorbent assays.^40^

The discovery of three new mosquito-specific flaviviruses brings the total number of these agents to 38 (Table 1). This illustrates the diversity of ISFs that may be present in the microbiome (virome) of mosquitoes. Undoubtedly, additional mosquito-specific flaviviruses exist and will be detected in the future. Based on the evidence to date,^1,24,30–35^ it appears that some of these ISFs have an effect on the vector competence of their mosquito hosts for related mosquito-transmitted flavivirus pathogens. Although the frequency of ISF infections in field populations of mosquitoes is currently unknown, it is probably relatively low; furthermore, the infection rate likely varies by locality and species. But the variety and number of ISFs (and EVEs) serves as yet another example that all *Ae. aegypti* or *Culex quinquefasciatus* do not have the same vector potential. Other recent studies have demonstrated that the gut microbiota (bacterial flora) of mosquitoes can also change the insect’s vector potential for some arboviruses by altering the mosquito’s basal innate immunity or by directly inhibiting the virus through bacterial metabolites.^41,42^

Although the effects of an insect’s microbiome on its vector competence are now being recognized by microbiologists and vector biologists, they apparently are not by most public health officials, epidemiologists, and modelers of arboviral diseases. It appears that the latter groups assume that all mosquitoes of a given species have the same vector potential and that it is possible to predict the risk, intensity of transmission, and spread of a disease caused by a mosquito-borne pathogen, such as dengue or Zika virus, based solely on climatic data, estimates of vector distribution and density, and susceptibility (immune status) of the local human population. But this approach is simplistic and misleading. The microbiome and resulting vector competence of a local *Ae. aegypti* population may be of equal importance in determining the character of an anticipated dengue or Zika outbreak, as the herd immunity of the local human population. Given the complexity of factors affecting vector competence of mosquitoes and other hematophagous insects, further research in this area is needed if we want to understand and control the transmission of vector-borne viral diseases.

In view of the similarity of some of the ISFs with the mosquito-borne flavivirus pathogens of vertebrates, one final consideration is whether one or more of the ISFs could evolve or mutate and acquire the ability to infect vertebrates. In other words, could an ISF like Marisma mosquito virus emerge sometime in the future as a human or animal pathogen? This is not a frivolous question. Recent metagenomics studies^43,44^ have shown that flavivirus-like and other negative-sense RNA viruses are much more numerous and diverse in invertebrates than in vertebrates, suggesting that the flavivirus pathogens may have evolved from earlier arthropod viruses. Thus it seems possible that an ISF could emerge as a vertebrate pathogen, although at present it is impossible to know how or when this might occur. However, this is another reason to discover, characterize, and monitor novel ISFs.
